# Patterns of triclosan resistance in Vibrionaceae

**DOI:** 10.7717/peerj.5170

**Published:** 2018-07-12

**Authors:** Keri A. Lydon, Megan J. Robertson, Erin K. Lipp

**Affiliations:** Department of Environmental Health Science, University of Georgia, Athens, GA, United States of America

**Keywords:** *Vibrio*, Antimicrobial resisance, Marine, Personal care products, Persistant contaminant

## Abstract

The antimicrobial additive triclosan has been used in personal care products widely across the globe for decades. Triclosan resistance has been noted among *Vibrio* spp., but reports have been anecdotal and the extent of phenotypic triclosan resistance across the Vibrionaceae family has not been established*.* Here, triclosan resistance was determined for Vibrionaceae strains across nine distinct clades. Minimum inhibitory concentrations (MIC) were determined for 70 isolates from clinical (*n* = 6) and environmental sources (*n* = 64); only two were susceptible to triclosan. The mean MIC for all resistant Vibrionaceae was 53 µg mL^−1^ (range 3.1–550 µg mL^−1^), but was significantly different between clades (*p* < 0.001). The highest mean triclosan MIC was observed in the Splendidus clade (200 µg mL^−1^; *n* = 3). Triclosan mean MICs were 68.8 µg mL^−1^ in the Damselae clade and 45.3 µg mL^−1^ in the Harveyi clade. The lowest mean MIC was observed in the Cholerae clade with 14.4 µg mL^−1^, which was primarily represented by clinical strains. There were no significant differences in triclosan MIC among individual species or among environmental strains isolated from different locations. Overall, phenotypic triclosan resistance appears to be widespread across multiple clades of Vibrionaceae.

## Introduction

The bacterial Family Vibrionaceae is made up of seven genera and 142 species, grouped into 23 distinct clades (Sawabe, Kita-Tsukamoto & Thompson, 2007; Sawabe et al., 2013). These aquatic bacteria exhibit high amounts of gene exchange (Polz et al., 2006), and act as opportunistic pathogens to both humans and marine organisms. Common pathogenic species in humans include *Vibrio cholerae*, *Vibrio vulnificus*, *Vibrio parahaemolyticus*, *Vibrio alginolyticus*, and *Photobacterium damselae*; however, any member of the Vibrionaceae associated with clinical disease is nationally reportable in the U.S. under the Cholera and Other *Vibrio* Illness Surveillance (COVIS) program (US Centers for Disease Control and Prevention (CDC), 2017). In some cases, these infections may lead to severe outcomes including fatal bouts of diarrhea due to dehydration or fatal septicemia in patients with compromised immune systems (Hlady & Klontz, 1996; Guerrant, Carneiro & Dillingham, 2003; Menon et al., 2014). Antibiotics are commonly used for treatment, with varying success rates (Wong et al., 2015). The role of antibiotic resistance in these bacteria has been investigated in environmental and retail oysters (Han et al., 2007), with some studies suggesting widespread resistance to multiple antibiotics in some strains of Vibrionaceae from both clinical and environmental origin (Garg et al., 2000; Baker-Austin et al., 2009; Kitaoka et al., 2011).

Although there is a well-established line of research on the role of antibiotic resistance among *Vibrio* spp., there is comparatively little research on resistance to non-therapeutic antimicrobial biocides in personal care products, which are used ubiquitously. One such antimicrobial is triclosan (2,4,4′-tricloro-2′–hydroxydiphenyl ether), for which resistance has been noted in some *Vibrio* spp., including *V. cholerae* (Massengo-Tiasse & Cronan, 2008; DeLorenzo et al., 2016). *V. cholerae* was shown to possess an alternate isoform (FabV) of FabI, the enoyl-acyl carrier protein reductase that triclosan targets through binding, leading to eventual inhibition of fatty acid synthesis (Levy et al., 1999; Reiss et al., 2002; Massengo-Tiasse & Cronan, 2008). FabV from *V. cholerae* was able to confer resistance to triclosan in *E. coli* clones with the alternate enzyme, with up to 20-fold higher concentrations (up to 10 µg mL^−1^) required to inhibit growth (Massengo-Tiasse & Cronan, 2008; Zhu et al., 2010). Other mechanisms of triclosan resistance that may be present include the expression of alternative or mutated enzymes, and/or the presence of efflux pumps, which has been noted in *Pseudomonas aeruginosa*, *E. coli*, and *Salmonella enterica* (Chuanchuen, Karkhoff-Schweizer & Schweizer, 2003; Braoudaki & Hilton, 2004; Yazdankhah et al., 2006). In fact, triclosan has been demonstrated to both induce antibiotic resistance in biofilms and benthic microbial communities (Nietch et al., 2013; Carey & McNamara, 2015) and confer cross-resistance to other important antimicrobials, as was found in *Salmonella* and *E. coli* (Braoudaki & Hilton, 2004). Understanding the extent of triclosan resistance in Vibrionaceae is important given that reported cases have increased by 54% since 2016 (Marder et al., 2018) and have nearly doubled since 1996 in the US (US Center for Disease Control and Prevention (CDC), 2016).

Research to date is limited to only a few studies but such evidence suggests that triclosan resistance may be common among *Vibrio* (Massengo-Tiasse & Cronan, 2008; DeLorenzo et al., 2016); however, there has been no attempt to systematically evaluate resistance patterns. Here, we examined triclosan resistance across a range of species and clades within Vibrionaceae, including environmental isolates, to provide a baseline for triclosan resistance in this group.

## Materials and Methods

### Vibrionaceae Isolates

Sixteen isolates (13 species representing five clades) were obtained from American Type Culture Collection (ATCC) or courteously provided from the culture collection of Rita Colwell (University of Maryland) ( Data S1). Members of clades represented are common human pathogens and include seven isolates of clinical origin. In addition, 54 presumptive Vibrionaceae isolates were collected from coastal surface waters on thiosulfate-citrate-bile salts-sucrose (TCBS) agar plates during a previous study (Lydon et al., 2017). Isolates were obtained from three coastal sites in the southeast U.S. between July and September 2014. Two stations were in the lower Florida Keys, including an offshore site (Looe Key Reef in the Florida Keys National Marine Sanctuary; 24.5449 N 81.40713 W; sampled under permit number FKNMS-2010-131-A1) and an inshore residential canal (Doctors Arm, Big Pine Key, FL; 24.700294 N 81.351825 W). The final station was Clam Bank Landing in North Inlet Estuary (Georgetown, SC; 33.333933 N 79.192913 W). Triclosan concentrations at the time of sampling for these locations were 103, 362, and 18 ng L^−1^, for Looe Key Reef, Doctors Arm Canal, and Clam Bank Landing, respectively (Lydon et al., 2017).

### Identification of environmental vibrionaceae isolates

Presumptive Vibrionaceae isolates were identified by sequencing of the Hsp60 gene (Thompson et al., 2005; Preheim, Timberlake & Polz, 2011; Jesser & Noble, 2018). Pure cultures were extracted by boiling lysis. Extracted DNA was quantified (NanoDrop 1000, Thermo Scientific, Wilmington, DE, USA) and diluted to 5 ng µL^−1^ before being subjected to PCR amplification using primers H279 (5′-GAATTCGAIIIIGCIGGIGA(TC)GGIACIACIAC-3′) and H280 (5′-CGCGGGATCC(TC)(TG)I(TC)(TG)ITCICC(AG)AAICCIGGIGC(TC)TT-3′) (Goh et al., 1996; Kwok et al., 2002; Thompson et al., 2005; Preheim, Timberlake & Polz, 2011). Amplification reactions contained 10 µL of 5x Master Mix (New England BioLabs (NEB), Ipswich, MA, USA; Final concentration 1×), 0.5 µM of each forward and reverse primers, and 10 µL of DNA template with molecular grade water for a final volume of 50 µL per reaction. PCR conditions on the thermal cycler (T100 Thermal Cycler, BioRad Laboratories Inc., Hercules, CA, USA) were 95 °C for 3 min for initial denaturation followed by 20 cycles of 30 sec at 95 °C, 30 sec at 37 °C, 1 min at 72 °C. The final extension step was 72 °C for 2 min. Amplicons were purified using equal volume SPRI magnetic beads (Sera-Mag SpeedBeads, Thermo Scientific, Fremont, CA, USA) (Rohland & Reich, 2012) on a 96-well magnetic plate (Promega MagnaBot II, Madison, WI, USA) and stored at −20 °C. Samples were sent to the Georgia Genomics and Bioinformatics Core (GGBC) (Athens, GA) and sequenced by Sanger sequencing (Applied Biosystems 3730xl 96-capillary DNA Analyzer, Foster City, CA, USA).

**Figure 1:**
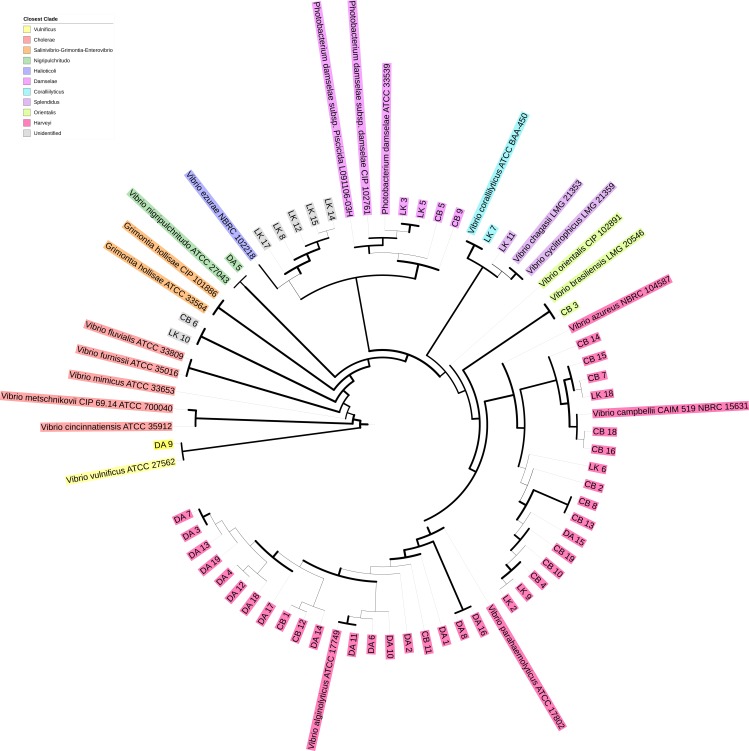
Phylogenetic tree of *hsp60* sequences of presumptive Vibrionaceae** isolates. Vibrionaceae with previously published *hsp60* sequences (cpnDB; http://cpndb.ca/; Accessed May 02, 2018) are indicated by their genus and specific epithet (accession numbers listed in Data S1). Branch thickness corresponds to aLRT SH-like branch support (thick line ≥ 0.5 (most support), intermediate line = 0.5, and thin line ≤ 0.5 (least support)) (Guindon et al., 2010).

To determine presumptive Vibrionaceae isolate identity, sequencing reads were loaded into Geneious (version R11) and checked for quality before being subjected to BLAST (Pearson & Lipman, 1988) against the chaperone database (cpnDB) by selecting for non-redundant group I sequences (*hsp60*) (http://cpndb.ca/; Accessed May 02, 2018). Subsequently, isolates were classified by clade and presumptive species by their closest cpnDB BLAST match. Isolate sequences were aligned with MUSCLE in Unipro UGENE v.1.26.3 (Edgar, 2004; Okonechnikov et al., 2012). The resulting alignment was used to build a phylogenetic tree using PhyML maximum likelihood with aLRT SH-like branch support (Guindon et al., 2010). The resulting tree was visualized using iTOL (version 4.0.3) and used in conjunction with BLAST match to identify closest Vibrionaceae clade (Letunic & Bork, 2007; Fig. 1). All sequences were submitted to NCBI GenBank with the BankIt submission tool (accession nos. MG975785–MG975835). The matching accession no. for each isolate can be found in the supplemental data file.

### Triclosan MIC screening

All isolates were screened for triclosan minimum inhibitory concentrations (MIC) using modified broth microdilution assays (CLSI, 2012). Isolates were grown overnight at 30 °C with shaking (130 rpm; Classic Series C-24 Incubator Shaker, New Brunswick Scientific, Edison, NJ, USA) in 2 mL of $ \frac{1}{2} $ strength Marine Broth (Zobell Marine Broth 2216, HiMedia, Mumbai, India) + NaCl (Final concentration 0.972%), after which 1 mL of growth was transferred to 4 mL of fresh Muller Hinton Broth + NaCl (Final concentration 2%) and continued to incubate at 30 °C for 4 h (with shaking at 130 rpm) to achieve log phase growth. *E. coli* (ATCC 15597) was also screened in the same manner as *Vibrio* isolates, but grown in LB broth in place of $ \frac{1}{2} $ strength Marine Broth. This isolate served as a triclosan positive control given that *E. coli* is susceptible to triclosan (McMurry, Oethinger & Levy, 1998; Lambert & Pearson, 2000)*.* Cultures were adjusted to 0.5 McFarland Standard in Muller Hinton Broth and exposed to eight triclosan concentrations, ranging from 0.78 µg mL^−1^ to 100 µg mL^−1^(in two-fold dilutions), in duplicate, with a final broth volume of 200 µL. Triclosan working stock (0.8 mg mL^−1^) was prepared by dissolving triclosan (Irgasan, ≥97.0% HPLC, Sigma Aldrich, St. Louis, MO, USA) into 100% ethanol (Decon Laboratories, King of Prussia, PA, USA). The final assay was conducted in UV-sterilized 96-well low binding plates (SiliGaurd, Analytical Sales and Services, Inc, Flanders, NJ, USA). In addition to triclosan exposures, each plate included two negative controls (sterile Muller Hinton Broth and 10% ethanol), as well as isolates without exposure to triclosan, in duplicate. The plates were placed at 120 rpm in a shaking incubator at 30°C and read at 48 h.

The lowest triclosan concentration in which there was no bacterial growth was noted as the MIC. The MIC concentrations for each replicate exposure were averaged. Those isolates that had MICs greater than 100 µg mL^−1^ were further screened at higher concentrations by streaking directly onto Muller Hinton Agar + 2% NaCl agar plates containing final triclosan concentrations of 0, 50, 100, 200, 300, 400, 500, 600, and 700 µg mL^−1^. All assays were completed in duplicate. Agar plates were used in place of broth due to the hydrophobic properties of triclosan and a tendency for triclosan to precipitate out of solution at higher concentrations.

Classification of susceptible and resistant bacteria by MIC value is normally determined by evaluation of an antibiotic’s properties including screening of MICs across a large subset of bacterial types (Sirot, Courvalin & Soussy, 1996). However, triclosan MIC data are limited and to our knowledge there is no published consensus on a cut-off level for resistant or susceptible MIC levels. Thus, we classified triclosan resistance in this study as any isolate exhibiting a triclosan MIC concentration higher than what was observed in our *E. coli* control, or greater than 3.1 µg mL^−1^.

### fabV in published vibrionaceae genomes

To investigate how widespread *fabV* was in published Vibrionaceae genomes, a nucleotide BLAST was conducted against the NCBI complete prokaryote genome database selecting only for those genomes classified as Vibrionaceae (taxid:641).

### Statistical analyses

Triclosan MIC readings were log-transformed to approximate a normal distribution. Results of the D’Agostino & Pearson normality test indicated that log-transformed data were normally distributed (K2 = 4.09, *p* = 0.1294). Triclosan readings were checked for the assumption of equal variances using the Brown-Forsythe test. Equal variances were found for all comparison types: by species (*F*_2,27_ = 0.2743, *p* = 0.7622), location (*F*_2,51_ = 0.42, *p* = 0.6558), and clade (*F*_3,47_ = 1.589, *p* = 0.2046). Having passed both assumptions of normality and equal variance, log-transformed triclosan MICs were compared using one-way ANOVA to determine differences amongst species, locations, and clades. For analysis of MIC by species, isolates were included in a species group if their BLAST closest match was greater than 97% identity ( Data S1) and there were more than three isolates available.

## Results

### Triclosan minimum inhibitory concentrations

**Figure 2:**
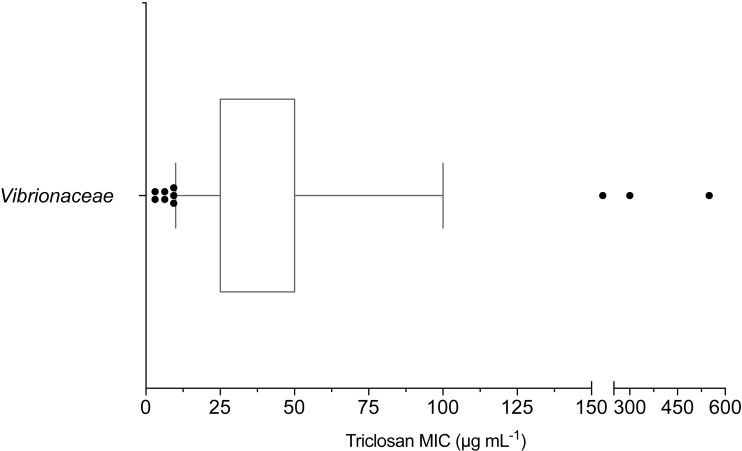
Box and whisker plot of triclosan MICs for Vibrionaceae isolates (*n* = 70). Whiskers indicate 10–90th percentile. *E. coli* was used as the control with an observed MIC of 3.1 µg mL^−1^.

The mean triclosan MIC observed for all Vibrionaceae isolates (*n* = 70) was 53 µg mL^−1^ with a range of 3.1–550 µg mL^−1^. Over 98% (69/70) of isolates showed phenotypic triclosan resistance (>3.25 µg mL^−1^) (Fig. 2). Only two Vibrionaceae isolates, both of which were collected from environmental sources, were susceptible to triclosan: *Photobacterium* sp*.* strain CB 6 (Damselae clade) from Clam Bank, SC and *Photobacterium* sp*.* strain DA 5 (Damselae clade) from Doctors Arm, FL. MICs of ≤100 µg mL^−1^ of triclosan were required to inhibit growth for 90% of isolates. There were 11 isolates that exhibited elevated levels of resistance (≥100 µg mL^−1^) to triclosan. These isolates belonged to both the *Photobacterium* and *Vibrio* genera and came from all three of our environmental sampling sites. The highest triclosan MIC (550 µg mL^−1^) was observed in *Vibrio* sp*.* strain LK 11 (closest clade was Splendidus; hsp60 sequence 90.30% identity with *Vibrio crassostreae* NZ_AJZC01000106) isolated from surface water at Looe Key Reef.

**Figure 3:**
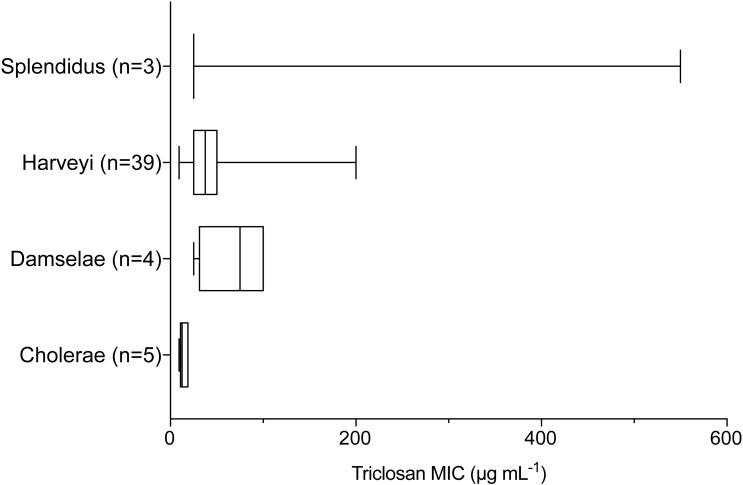
Triclosan MIC by clade, where there were greater than three isolates per clade. Results of one-way ANOVA demonstrated significant differences in mean rank triclosan MICs for isolates by clade (*F*_3,47_ = 4.615, *p* = 0.0178). Tukey’s post-hoc multiple comparisons tests indicated the Cholerae clade had significantly lower triclosan MICs than the Damselae (adjusted *p*-value = 0.0178), Splendidus (adjusted *p*-value = 0.0149), and Harveyi (adjusted *p*-value = 0.0283) clades.

Triclosan MICs were compared by clade for those clades that included more than three isolates. Results of one-way ANOVA indicated significant differences in mean triclosan MICs for the Cholerae, Damselae, Harveyi, and Splendidus clades (*F*_3,47_ = 4.615, *p* = 0.0065). Mean MICs were 14.4 µg mL^−1^, 68.8 µg mL^−1^, 45.3 µg mL^−1^, and 200 µg mL^−1^ for the Cholerae (*n* = 5), Damselae (*n* = 4), Harveyi (*n* = 39), and Splendidus (*n* = 3) clades, respectively (Fig. 3). Post-hoc multiple comparisons (Tukey’s Multiple Comparisons Test) indicated significantly lower triclosan MICs for the Cholerae clade in comparison with Harveyi (adjusted *p*-value = 0. 0283), Splendidus (adjusted *p*-value = 0. 0149), and Damselae clades (adjusted *p*-value = 0.0178). Of these clades, Splendidus exhibited the widest range of triclosan MIC readings (25-550 µg mL^−1^; *n* = 3) while Cholerae, primarily from clinical sources, showed the smallest range (3.1–18.8 µg mL^−1^; *n* = 5).

**Figure 4:**
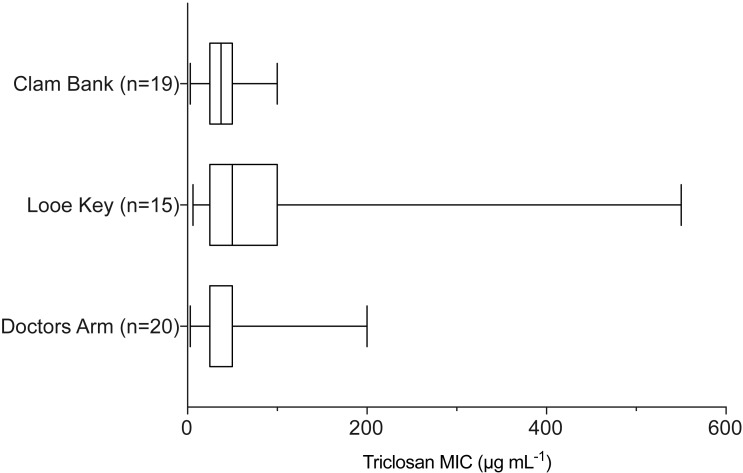
Triclosan MIC by location sampled (Doctors Arm, Looe Key, and Clambank Landing). Results of one-way ANOVA showed no significant differences in triclosan MICs for *Vibrionaceae* by location sampled (*F*_2,51_ = 1.346, *p* = 0.2694).

**Figure 5:**
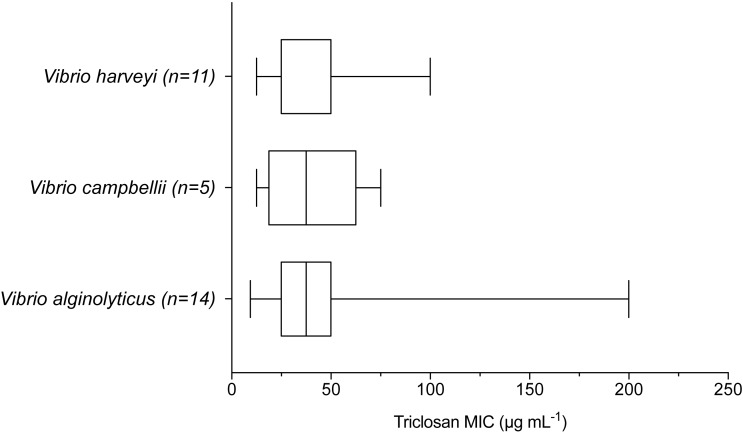
Triclosan MIC by species within the Harveyi clade where there were greater than three isolates per species tested. Results of one-way ANOVA showed no significant differences (*p* > 0.05) in triclosan MICs among these species (*F*_2,27_ = 0.2448, *p* = 0.7846).

When examining environmental isolates based on where they were obtained, there were no significant differences in mean triclosan MICs among the three stations (Fig. 4; *F*_2,51_ = 1.346, *p* = 0.2694). There were no significant differences in triclosan MICs for environmental Vibrionaceae among presumptive species in the Harveyi Clade (when there were more than three isolates per species; clinical isolates excluded) (*F*_2,27_ = 0.2448, *p* = 0.7846). Mean triclosan MICs were 54 µg mL^−1^ for *V. alginolyticus* (*n* = 14), 40 µg mL^−1^ for *Vibria brasiliensis* (*n* = 5), 37.5 µg mL^−1^ for *Vibria harveyi* (*n* = 11) (Fig. 5). Of these presumptive species, *V. alginolyticus* exhibited the widest range of triclosan MIC readings (9.4–200 µg mL^−1^), while the smallest range was observed in *V. campbelli* (12.5–75 µg mL^−1^).

Investigation of *fabV* in whole genome sequences of Vibrionaceae resulted in 119 significant hits for the *fabV* gene within 131 published closed Vibrionaceae genomes. This demonstrated that *fabV* typically occurs in Vibrionaceae but is not universal. In addition, *fabV* appeared to occur on either chromosome I or II, though occurring most frequently on chromosome I.

## Discussion

This study characterized phenotypic triclosan resistance in a wide range of Vibrionaceae isolates through measurement of MICs. Results suggest that members of Vibrionaceae are broadly resistant to triclosan across three genera (*Vibrio, Allivibrio*, and *Photobacterium*), nine clades (Cholerae, Coralliilyticus, Damselae, Fischeri, Halioticoli, Harveyi, Orientalis, Splendidus, and Vulnificus), and 15 species (*Aliivibrio fischeri*, *V. alginolyticus, Vibrio brasiliensis, V. campbellii*, *V. cholerae*, *Vibrio coralliilyticus*, *Vibrio furnissii*, *V. harveyi*, *Vibrio mimicus*, *V. parahaemolyticus*, *Vibrio pelgaius, Vibrio rotiferianus, Vibrio splendidus*, *V. vulnificus, and P. damselae*). Furthermore, these included known pathogens (e.g., clinical strains from established culture collections), as well as recently collected environmental isolates from distinctly different ecosystems with variable amounts of anthropogenic impacts (Lydon et al., 2017).

Triclosan resistance was previously documented in *V. cholerae* (Massengo-Tiasse & Cronan, 2008) and was suspected in unidentified *Vibrio* spp. from the estuarine environment (DeLorenzo et al., 2016), but resistance had not been systematically examined across a wide range of Vibrionaceae. Moreover, to our knowledge, this is the first study to report phenotypic triclosan resistance in the closely related genus *Photobacterium*, which include species that are both human and fish pathogens (e.g., *P. damselae*) (Rivas, Lemos & Osorio, 2013). We also note that the highest triclosan resistance MIC was observed in a presumptive *Vibrio crassostreae* isolate, an emerging shellfish pathogen (Bruto et al., 2017). Triclosan MICs demonstrated here for Vibrionaceae are consistent with MICs reported for known triclosan resistant bacteria, including *Pseudomonas aeruginosa* and *Staphylococcus aureus* (MICs ranging from 5 to greater than 2,000 µg mL^−1^) (Chuanchuen, Karkhoff-Schweizer & Schweizer, 2003; Zhu et al., 2010).

There is growing evidence that *Vibrio* could be enriched in coastal waters by pharmaceutical wastes and antimicrobial agents in personal care products (Peele et al., 1981; Grimes, Singleton & Colwell, 1984; DeLorenzo et al., 2016; Lydon et al., 2017). Triclosan resistance has specifically been hypothesized as a potential factor leading to increased abundance of *Vibrio* bacteria after exposure of seawater or marine organisms to triclosan (DeLorenzo et al., 2016; Lydon et al., 2017). *V. alginolyticus* demonstrated MICs up to 200 µg mL^−1^ and are an emerging cause of vibriosis cases in the US, with infections increasing by 12-fold from 1996 to 2012, the highest rate of any *Vibrio* (Slifka, Newton & Mahon, 2017). Triclosan has the capacity to induce cross- or multiple- antibiotic resistance (Nietch et al., 2013; Carey & McNamara, 2015); therefore, we suspect there are risks of inducing resistance in the environment as Vibrionaceae have plastic genomes and high gene transfer rates (Polz et al., 2006; Quirke et al., 2006).

We observed significant differences in MIC between *Vibrio* at the Clade level, suggesting that there may be some evolutionary role in resistance; however, much of this difference was driven by low MICs among the Cholerae clade, which was over-represented by clinical strains compared to other clades. Furthermore, differences were not observed between species, which may support the alternate hypothesis that triclosan resistance may be ubiquitous among *Vibrio* but may occur through multiple mechanisms. Triclosan resistance has previously been identified in *V. cholerae* through the expression of a FabI analog, FabV, which prohibits triclosan from binding and interrupting fatty acid synthesis (Massengo-Tiasse & Cronan, 2008). Analysis of published closed whole genome sequences confirmed that *fabV* is highly associated with *Vibrio* spp., including non-cholerae species, but also demonstrated that is it not universal among Vibrionaceae genomes. We suspect the wide variability in triclosan resistance for Vibrionaceae is due to the presence of multiple resistance pathways, including the presence of FabV, overexpression of FabI, and efflux pumps that may be acting singularly or synergistically. These are particularly important as they could confer resistance to other antibiotics, which could have significance for clinical applications.

## Conclusions

Triclosan resistance appears to be nearly universal across Vibrionaceae isolates tested in this study. Levels of resistance (MICs) were distinguishable between Vibrionaceae clades, suggesting that some level of resistance is dependent on phylogeny, rather than environmental source, which showed no difference in MICs. This work is important in that it provides a broad examination of phenotypic resistance to this persistent antimicrobial compound among this group of potential pathogens. However, future investigations are needed to establish why triclosan resistance is so variable across the Vibrionaceae. We suspect the wide variability is likely due to multiple resistance mechanisms, which could have broader implications for emergence of antibiotic resistance.

##  Supplemental Information

10.7717/peerj.5170/supp-1 Data S1 MIC raw data and sequence accession numbers Tabs in this data file include: isolate source, MIC data, and assigned accession numbers (tab 1), hsp60 BLAST identification results (tab 2), and type strain accession numbers used to build hsp60 tree (tab 3).

10.7717/peerj.5170/supp-1 Supplemental Information 1 Sanger sequences of the hsp60 gene for characterization of Vibrionaceae isolates

## Additional Information and Declarations

### Field Study Permissions

The following information was supplied relating to field study approvals (i.e., approving body and any reference numbers): At Looe Key Reef collections were all made under NOAA permit FKNMS-2010-131-A1.

### DNA Deposition

The following information was supplied regarding the deposition of DNA sequences: The sequences described here are accessible via GenBank accession numbers MG975785–MG975835. Sequence data can also be found in the Supplemental Information.

### Data Availability

The following information was supplied regarding data availability: The raw data are provided in a Supplemental File.
